# D-ärztlich veranlasste Psychotherapie: Wichtiger Baustein im Heilverfahren der gesetzlichen Unfallversicherung

**DOI:** 10.1007/s00113-023-01313-0

**Published:** 2023-03-14

**Authors:** Josephine Jugert, Stefan G. Schröder, Claudia Drechsel-Schlund, Peter Angerer

**Affiliations:** 1 Klinik für Innere Medizin, KMG Klinikum Güstrow, Friedrich-Trendelenburg-Allee 1, 18273 Güstrow, Deutschland; 2 Institut für Arbeits‑, Sozial- und Umweltmedizin, Heinrich-Heine-Universität Düsseldorf, Düsseldorf, Deutschland; 3 Klinik für Psychiatrie und Psychotherapie, KMG Klinikum Güstrow, Friedrich-Trendelenburg-Allee 1, 18273 Güstrow, Deutschland; 4 Berufsgenossenschaft für Gesundheitsdienst und Wohlfahrtspflege (BGW), Hamburg, Deutschland

**Keywords:** Arbeitsunfälle, DGUV, Traumafolgestörungen, Psychotherapeutenverfahren, Posttraumatische Belastungsstörung, Work-related injuries, Posttraumatic stress disorder, Psychotraumatology, Rehabilitation, Psychological sequelae of accidents

## Abstract

**Hintergrund:**

Deutschlandweit liegt mit etwa 1 Mio. pro Jahr die Zahl von Arbeits- und Wegeunfällen seit Jahren konstant hoch, aktuell allerdings pandemiebedingt niedriger, aufgrund von partiellen Betriebsschließungen, Lockdown-Maßnahmen und der Zunahme von Arbeit aus dem „Homeoffice“.

**Fragestellung:**

Die Deutsche Gesetzliche Unfallversicherung e. V. (DGUV; gemeinsamer Spitzenverband der Unfallversicherungsträger) hat 2012 mit dem sog. Psychotherapeutenverfahren ein Instrument zur Förderung und zur Regulierung fachkundiger Versorgung psychischer Unfallfolgen geschaffen. Wie ist diese Regelung angenommen worden? Welche Fallkonstellationen werden beobachtet?

**Material und Methode:**

Nationale Daten zur Nutzung des Psychotherapeutenverfahrens bei Arbeits- und Wegeunfällen werden rückblickend für die Jahrgänge 2013–2021 vorgestellt, exemplarisch auch regionale, inklusive Fallskizzen (aus Güstrow).

**Ergebnisse:**

Deutschlandweit hat sich im betrachteten Zeitraum der Prozentsatz der Psychotherapien von 0,47 auf 0,96 % verdoppelt.

**Diskussion:**

Die 10-Jahresbilanz ist positiv, das Psychotherapeutenverfahren wird offensichtlich gut angenommen. Der tatsächliche Psychotherapiebedarf lässt sich nur aus Einzelstudien abschätzen, repräsentative Studien fehlen. Interdisziplinäres klinisches und wissenschaftliches Engagement für die Psychotraumatisierten ist notwendig und zu optimieren. Grundwissen in Psychotraumatologie ist daher bereits für die D‑Arzt-Medizin in den unfallchirurgischen Weiterbildungskatalog aufgenommen worden.

## Abstract (en)

**Background:**

In Germany around 1 Million working and commuting accidents per year are officially registered. Since several years this number is constantly at this high level, only just since the appearance of the actual Covid pandemia significantly lower because of lockdowns and increase of homeoffice workplaces.

**Objective:**

To ensure the professional care of psychotraumatic sequelae of work-related injuries, in 2012 new regulations were introduced by the DGUV, the national umbrella organization of the German statutory health insurances. This healthcare reform (named “Psychotherapeutenverfahren”, verbally translated: psychotherapists’ procedure) is looked at 10 years later. Is the new regulation accepted by the surgeons’ community? What case constellations can be observed?

**Material and methods:**

The use of this new instrument among trauma surgeons was evaluated by national data for the complete years 2013–2021. Additionally, we include regional data and case reports from a multidisciplinary medical facility including trauma surgery and psychiatry, located in Güstrow (Northeast Germany).

**Results:**

Nationwide the percentage of psychotherapy amongst the regular treatment has doubled between 2013 and 2021 from 0.47 to 0.96%.

**Discussion:**

Looking at the first 10 years of its clinical implementation, the new German psychotherapy regulation is apparently well accepted by the accident insurance consultants. As data from population based studies are not available, the actual percentage of psychological sequelae of accidents in this cases is not known yet. So there is further need of research and training in this interdisciplinary field to ensure appropriate treatment of victims of work-related accidents. The integration of psychotraumatological facts in the educational agendas of accident insurance consultants has already started.

**Graphic abstract:**

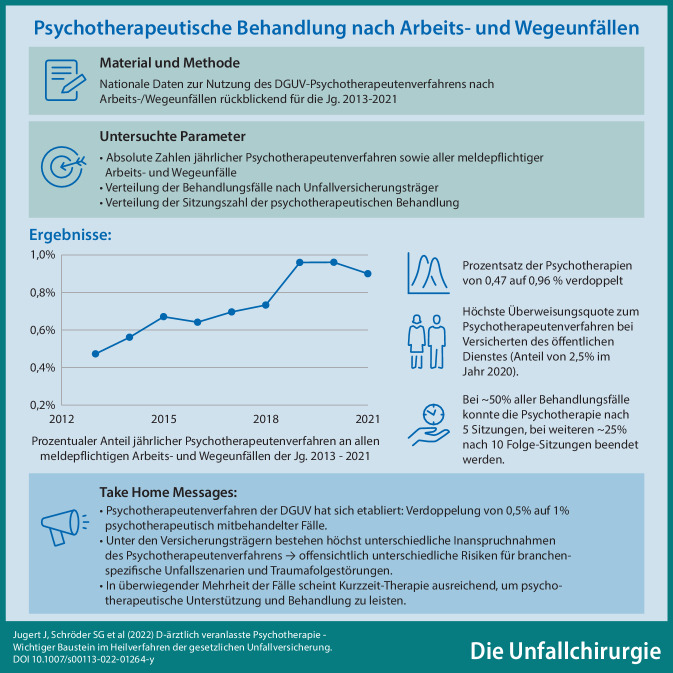

## D-ärztlicher und psychotraumatologischer Hintergrund

In Deutschland werden jährlich etwa *1* *Mio.* meldepflichtige Arbeits- und Wegeunfälle erfasst, wobei diese Zahl in den letzten Jahren in etwa konstant blieb, 2020/2021 allerdings pandemiebedingt niedriger lag. Nicht selten kommt es dabei nicht nur zu einem unfallchirurgischen, sondern zusätzlich auch zu einem psychischen Trauma (teilweise sogar ausschließlich). Daher hat die Deutsche Gesetzliche Unfallversicherung e.V. (*DGUV*, gemeinsamer Spitzenverband der Unfallversicherungsträger) Mitte 2012 das sog. *Psychotherapeutenverfahren* eingeführt, das die D‑ärztliche Weiterverweisung erleichtert und reguliert. Unpublizierte DGUV-Daten für das gesamte Bundesgebiet werden nachfolgend zugänglich gemacht und ein beispielhafter Einblick in die psychiatrisch-psychotherapeutische Behandlungspraxis eines Klinikums der Grund- und Regelversorgung mit psychiatrischer Instituts- und D‑ärztlicher Ambulanz gegeben (KMG Klinikum Güstrow).

## Gute Akzeptanz des Psychotherapeutenverfahrens

Die Unfallversicherungsträger (UV-Träger) haben dem Bundesministerium für Arbeit und Soziales (BMAS) alljährlich über das Unfallgeschehen zu berichten. Die statistischen Daten zu den *meldepflichtigen Arbeits- und Wegeunfällen* (mit einer Arbeitsunfähigkeit über 3 Tagen) sind allgemein zugänglich (https://publikationen.dguv.de).

**Figure 1:**
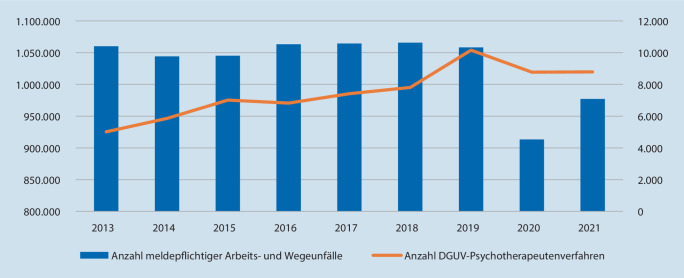

Die DGUV-*Psychotherapie*-Daten für die Jahrgänge 2013–2021 werden in der vorliegenden Übersicht nach punktuellen Mitteilungen [12–14] hier *erstmals* systematisch zugänglich gemacht. Im genannten 9‑Jahres-Zeitraum hat sich demzufolge der Anteil derjenigen Verunfallten *verdoppelt*, die zu psychotherapeutischer Mit- bzw. Weiterbehandlung überwiesen worden sind, nämlich von *0,47* *%* im Jahre 2013 auf *0,96* *%* im Jahre 2019. Somit wurde *2019* erstmals die Zahl von *10.000* DGUV-Psychotherapeutenverfahren überschritten (Abb. 1). Das Verfahren wird also offensichtlich gut angenommen, von D‑ärztlicher, psychotherapeutischer und nicht zuletzt auch von Versichertenseite. Die Praxis der letzten Jahre zeigte dabei, dass in über 80 % der Fälle der psychotherapeutische Behandlungsimpuls vom UV-Träger ausgeht. Die unmittelbare Überweisung durch den D‑Arzt (§ 12 des Vertrages Ärzte/UV-Träger) findet deutlich seltener statt.

**Figure 2:**
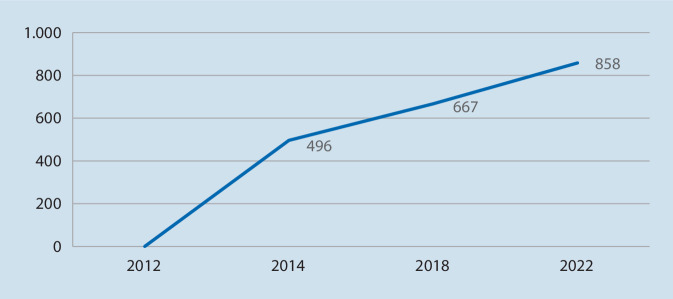

Es ist sogar von einer noch größeren Zahl psychotherapeutischer Fälle auszugehen; die Daten beziehen sich nur auf abgeschlossene Behandlungsfälle, somit sind Patienten und Patientinnen mit langwieriger, noch laufender Therapie nicht berücksichtigt und dementsprechend unterrepräsentiert [12]. Zudem werden nur Mitteilungen von Psychotherapeutinnen und Psychotherapeuten statistisch datiert, welche im Netzwerk der DGUV aufgenommen sind. Die UV-Träger nehmen jedoch auch außerhalb dieses Netzwerks erfolgreich Kooperationen zur psychotherapeutischen Behandlung vor, welche numerisch nicht erfasst werden. In den letzten Jahren wurde das *Psychotherapeutennetzwerk* jedoch weiter* ausgebaut*. So hat sich die Anzahl gelisteter Netzwerkpartner nahezu verdoppelt, von 496 im Jahr 2014 auf zuletzt 858 Netzwerkpartner im Jahr 2022 (Abb. 2).

**Figure 3:**
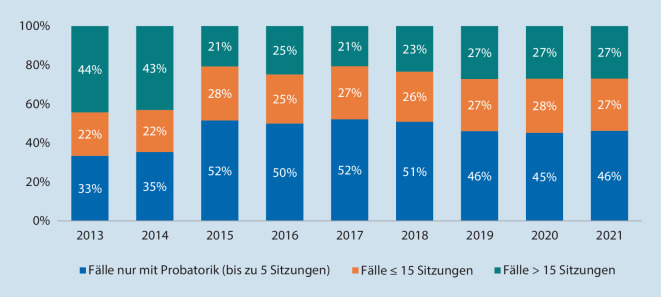

Nach Einleitung des Psychotherapeutenverfahrens stehen dem Patienten unabhängig von einer gesicherten Kausalität und ohne formales Beantragungsverfahren *5 probatorische Sitzungen* zu. Bei anhaltendem Therapiebedarf können nach entsprechendem Antrag und Prüfung durch den UV-Träger weitere 10 Sitzungen bewilligt werden, falls notwendig sind auch längere Therapieeinheiten möglich [12, 13]. Die statistische Erfassung der abgeschlossenen Behandlungsfälle konnte hierzu belegen, dass in den letzten Jahren in ca. der Hälfte aller behandelten Fälle nach den probatorischen Sitzungen und bei ca. 25 % nach weiteren 10 Sitzungen die Therapie beendet werden konnte (Abb. 3).

**Figure 4:**
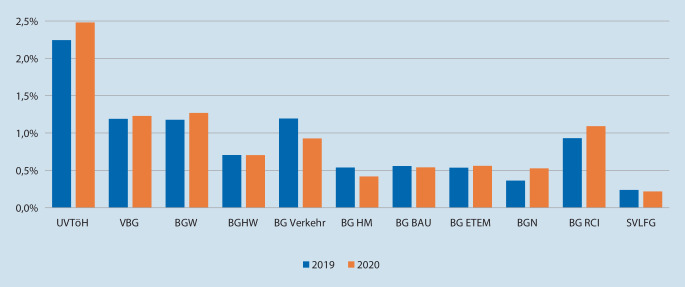

Je nach Berufsfeld bestehen unterschiedlich hohe Risiken für Unfallgeschehen, Bedrohungs- bzw. Gewaltereignisse oder Konfrontation mit Extremsituationen. Als besonders gefährdete Berufsgruppen sind u. a. *Dachdecker, Rettungs*- und *Pflegekräfte, Wach- und Sicherheits‑, Polizei*- und *Bundeswehrbedienstete, Lokführer* oder auch Angestellte im *Einzelhandel* zu nennen [7, 21]. Dabei können je nach Branche v. a. *rein psychische Traumata*, also gänzlich ohne körperliche Verletzungen, auftreten. Es zeigen sich auch in den jeweiligen Fallzahlen der UV-Träger im DGUV-Psychotherapeutenverfahren große Diskrepanzen. Exemplarisch für die Jahre 2019 und 2020 wurden die prozentualen Verhältnisse zu allen meldepflichtigen Arbeits- und Wegeunfällen und der psychotherapeutischen Inanspruchnahme der jeweiligen UV-Träger dargelegt (Abb. 4). Die höchste Überweisungsquote zum Psychotherapeutenverfahren nach einem Arbeits- oder Wegeunfall ist demnach beim *Unfallversicherungsträger der öffentlichen Hand* (UVTöH) zu verzeichnen (2,5 %).

## Der psychotherapeutische Bedarf ist damit noch nicht gedeckt

Der tatsächliche Psychotherapie*bedarf* nach Arbeits- und Wegeunfällen ist mangels guter Daten hierzu nicht exakt zu evaluieren [3], liegt jedoch mit Sicherheit *höher als* bei oben genannten *1* *%*, die jetzt erreichte Psychotherapiequote. Je nach betrachtetem unfallchirurgischem Kollektiv dürfte der psychisch traumatisierte Anteil der Verletzten unterschiedlich hoch ausfallen. Die Mehrheit der Studien bezieht sich ausschließlich auf Kollektive mit initialem unfallchirurgisch-**stationärem** Behandlungsbedarf (also unfallchirurgisch schwerere Fälle und keine Bagatelltraumata), wie beispielsweise auch die mehrteilige **F**reiburger **A**rbeits**u**nfall**st**udie (*FAUST*, [4–6, 19]). Andere Studien untersuchten die Prävalenzen von Traumafolgestörungen je nach Berufsgruppen [18] oder der Art des traumatischen Ereignisses [15, 20]. Seelische Folgen von Unfällen treten somit gemäß systematischer Forschung [7] sowie klinischer Einschätzung [22] in der Größenordnung von *10–30* *%* auf, sodass aktuell allenfalls ein Zehntel der *zusätzlich oder rein* psychisch Traumatisierten fachspezifisch (psychiatrisch-psychotherapeutisch) mit- bzw. weiterbehandelt werden würde.

Vor allem im Bereich der rein psychischen Traumata kann eine *hohe Dunkelziffer* vermutet werden. Eine Meldung von Arbeits- und Wegeunfällen ist verpflichtend bei einer Arbeitsunfähigkeit von mehr als 3 Tagen. Aufgrund fehlender körperlicher Verletzungen und oftmals vorhandener Arbeitsfähigkeit wird häufig keine Meldung an den UV-Träger vorgenommen [12]. Dabei gilt auch die Beobachtung eines Unfallereignisses (z. B. Zeuge einer tödlichen Verletzung eines Kollegen, Überfahrtraumen bei Zugführer) mit rein psychischen Unfallfolgen als Arbeitsunfall und kann eine Arbeitsunfähigkeit sowie eine (teils sogar isolierte) psychiatrische Behandlung notwendig machen. Häufig unterbleibt auch im Nachgang bei zeitlich verzögerter Entwicklung von psychischen Folgestörungen eine Meldung an den UV-Träger, weil oftmals die Konsultation zunächst beim Hausarzt erfolgt und somit keine gezielte Therapie über die UV-Träger eingeleitet werden kann [12]. Die UV-Träger empfehlen aus diesem Grund auch dann eine vorsorgliche Meldung, wenn eine Arbeitsunfähigkeit unter 3 Tagen vorliegt. Somit können diese auch zu einem späteren Zeitpunkt proaktiv im Sinne einer Frühintervention sowie zur Diagnoseklärung tätig werden oder um ein niederschwelliges Unterstützungsangebot zu unterbreiten.

Ebenso ist bei körperlich Verletzten nach einem Arbeits- oder Wegeunfall anzunehmen, dass aufgrund der oftmals langwierigen Therapiemaßnahmen die physischen Verletzungen im Vordergrund stehen und so psychische Traumafolgestörungen verzögert oder gar nicht erkannt werden [17].

Zu berücksichtigen ist: Der D‑Arzt gibt Hinweise zu psychischen Symptomen im Erst- oder im Verlaufsbericht, in der Mehrzahl der Fälle (nach eigener Schätzung über 80 %), aber *die UV-Träger erteilen selbst den Behandlungsauftrag* an die Psychotherapeuten. Nur bei einem kleinen Anteil findet eine Hinzuziehung (Überweisung zur Mitbehandlung) unmittelbar durch den D‑Arzt statt.

## Woran erkennt man den Psychotherapiebedarf?

Der unfallchirurgisch spezialisierte D‑Arzt ist zu seiner Unterstützung im eng getakteten Klinik- und Praxisalltag auf praxisorientierte Empfehlungen von psychiatrisch-psychotherapeutischer Seite angewiesen [1], um rechtzeitig auf Warnsymptome einer psychischen Traumafolgestörung reagieren und nach dem DGUV-Psychotherapeutenverfahren als „*Lotse*“ fachspezifische Mitbehandlung einleiten zu können [21]. Spezifisch sind die von der DGUV in einer Informationsschrift für den D‑Arzt publizierten, für die D‑ärztliche Sprechstunde u. E. nützlichen, folgenden *5 Fallkonstellationen* [11]:

1. **psychische Traumen**
  z. B. Raubüberfälle, Miterleben oder Herbeiführen eines tödlichen oder schweren Unfallgeschehens,
2. **psychische Gesundheitsstörungen im Zusammenhang mit Schwerstverletzungen**
  z. B. Polytraumatisierung, Querschnittslähmung, Brandverletzung,
3. **Fälle mit körperlichen Verletzungen und Hinweisen auf psychische Symptome**
  z. B. Schlafstörungen, Ängste, Vermeidungsverhalten, Niedergeschlagenheit und Rückzugsverhalten,
4. **Fälle mit auffälligen Krankheitssymptomen**
  z. B. Ausweitung des Beschwerdebildes, Diskrepanz zwischen objektivierbarem Befund und subjektivem Beschwerdebild, Überschreitung der zu erwartenden Arbeitsunfähigkeitsdauer,
5. **Fälle mit Belastungsfaktoren, die sich negativ auf die Unfallverarbeitung auswirken**
  z. B. Verlust des Arbeitsplatzes, Pflege von Angehörigen.

Als *prädiktiv* für das Auftreten einer psychoreaktiven Störung werden nach einigen Risikofaktorenstudien u. a. *weibliches* Geschlecht, *jüngeres und älteres* Lebensalter, *Verletzungsschwere* sowie eine ausgeprägte *psychische Initialsymptomatik* angesehen [5, 10, 23]; andere Arbeitsgruppen [8, 9] konnten dieses Risikoprofil jedoch *nicht* replizieren, sodass hier noch interdisziplinärer Forschungsbedarf besteht. Als *protektiver* Faktor gilt soziale Unterstützung durch das private und/oder soziale Umfeld, insbesondere auch durch den Arbeitgeber. Vorangegangene Traumata sowie psychische Vorerkrankungen *steigern* hingegen das Risiko einer psychotraumatologischen Störung [7, 16].

Stärker als der objektive unfallchirurgische Traumaschweregrad (z. B. gemäß ISS, Injury Severity Scale) scheint das *subjektive* Erleben des Traumas als lebensbedrohlich die psychische Traumatisierung vorherzusagen [16]. Die meisten psychoreaktiven Störungen treten nur *passager* auf, in einigen Fällen können diese jedoch unbehandelt *chronifizieren* [3]. Die Prognose einer psychoreaktiven Störung nach einem Unfall hängt wesentlich davon ab, dass die psychotherapeutische Behandlung von D‑ärztlicher Seite *frühzeitig* eingeleitet wird. Vor allem Patientinnen und Patienten mit ausgeprägter psychischer Initialsymptomatik profitieren von einer frühzeitigen Überweisung; eine langfristige Symptomreduktion lässt sich so erzielen [4]. Nicht selten zeigen sich jedoch nicht sogleich, sondern *erst im Verlauf *der nachfolgenden Tage und Wochen Hinweise auf eine manifeste Traumafolgestörung. Daher wird ein Langzeit-Monitoring des psychopathologischen Befunds gefordert [2, 4]. Dies ist für den unfallchirurgisch spezialisierten D‑Arzt allerdings u. E. definitiv nicht zu leisten.

Nach Empfehlungen der *S2k-Leitlinie „Diagnostik und Behandlung von akuten Folgen psychischer Traumatisierung“ *[7] sollten Betroffene nach traumatischen Ereignissen *innerhalb weniger Stunden bis Tage* eine psychotherapeutische Behandlung angeboten bekommen. Dabei ist die *Aufklärung *über mögliche psychische Unfallfolgen als eine* häufig auftretende Reaktion,* welche sich auch zeitlich verzögert entwickeln können, *Bewältigungsstrategien* sowie weitere *Therapiemöglichkeiten* von großer Bedeutung. Bereits Informationen darüber können Betroffene bei der Unfallverarbeitung unterstützen und Belastungssymptome reduzieren. Das Angebot einer psychotherapeutischen Mitbehandlung beruht dabei auf *Freiwilligkeit*, sollte jedoch schon in diesem Rahmen frühzeitig erfolgen.

Die genannte sowie die *S3-Leitlinie „Posttraumatische Belastungsstörung“* [17] sind im Internet frei zugänglich und geben umfassend den aktuellen Kenntnis- und Forschungsstand sowie handlungsleitende Empfehlungen wieder.

## Regionales Beispiel: ein Einblick in die psychotherapeutische Behandlung aus dem KMG Klinikum Güstrow

Die Barlachstadt Güstrow liegt im Flächenland Mecklenburg-Vorpommern und ist Kreisstadt für den Landkreis Rostock. Das betrachtete 500-Betten-Klinikum (mit Luftrettungsstützpunkt) verfügt sowohl über eine D‑Arzt-Sprechstunde als auch über eine psychiatrische Institutsambulanz (PIA), die einer bettenführenden Abteilungspsychiatrie mit Versorgungsauftrag angegliedert ist.

Im Durchschnitt wurden in den letzten 5 Jahren in der D‑ärztlichen Sprechstunde rund 1000 volljährige Behandlungsfälle (überwiegend *ambulant* behandelt) jährlich erfasst und davon ca. 3 bis 4 Fälle D‑ärztlich zur ambulanten psychiatrischen Mitbehandlung hausintern überwiesen. Die Therapie erfolgte dabei in Kooperation mit der DGUV außerhalb des Psychotherapeutennetzwerks, ist jedoch in analoger Anwendung des oben genannten DGUV-Psychotherapeutenverfahrens zu betrachten.

Um einen genaueren Überblick zu erhalten, welche Patientinnen und Patienten nach erlebtem Arbeits- oder Wegeunfall in eine psychotherapeutische Behandlung überwiesen worden sind, und inwiefern einzelne Faktoren eine Rolle spielen könnten, wurden Güstrower Daten der Jahrgänge 2000–2020 retrospektiv analysiert. Im genannten Zeitraum wurden insgesamt 48 volljährige Unfallopfer D‑ärztlich nach einem Arbeits- oder Wegeunfall in die psychiatrische Institutsambulanz überwiesen (alle zur ambulanten Mitbehandlung).

Soziodemografisch zeigte sich in der psychiatrisch-überwiesenen D‑ärztlichen Kohorte ein *ausgeglichenes Geschlechtsverhältnis* mit *einem mittleren Alter von 44 Jahren*. Fast *jeder Fünfte* der Kohorte (17 %) wies eine *psychiatrische Vorerkrankung *auf. Zu *je 50* *%* wurde nach einem *Wege- bzw. Arbeitsunfall* überwiesen. Die Mehrheit der Kohorte (77 %) erlitt nur *ein leichtes bis kein physisches Trauma* (ISS < 9). Die häufigsten psychiatrischen Hauptdiagnosen waren die *posttraumatische Belastungsstörung* (40 %), *Anpassungsstörungen* (35 %) und die *akute Belastungsreaktion* (15 %). Ähnlich zum Bundesdurchschnitt der DGUV konnte mit rund 60 % der Behandlungsfälle die Therapie innerhalb der probatorischen Sitzungen beendet werden, weitere 20 % nach 10 zusätzlichen Sitzungen.

In fast allen Fällen wurden als eines der vordergründig bestehenden Symptome *Schlafstörungen* genannt (94 %), nachfolgend *depressive Verstimmung* (79 %), *Albträume* (46 %) sowie *Grübelneigung* (42 %) (Tab. 1).

**Table 1:** 

Initial berichtete Symptome	Prozentualer Anteil der Patienten (%)
Schlafstörungen	94
Depressive Verstimmung	79
Albträume	46
Grübelneigung	42
Spezifische/isolierte Ängste	38
Flashbacks	33
Konzentrationsschwierigkeiten	21
Sozialer Rückzug	21
Vermeidungsverhalten	15
Panikattacken	10
Schädlicher Gebrauch von Alkohol	8

Unter Verwendung der von der DGUV veröffentlichten typischen 5 Fallkonstellationen (s. oben) für eine psychiatrisch-psychotherapeutische Überweisung wurden die Patientinnen und Patienten der Güstrower Kohorte in psychiatrisch-fachärztlicher Unterstützung zu der jeweilig treffendsten Fallkonstellation zugeordnet und eine vertiefende Analyse vorgenommen:

### Psychische Traumen

- Mit rund 35 % der größte Anteil in der Kohorte,
- *Bei rund 77* *% der Fälle bestand ein Fremdtrauma*, also das Miterleben bzw. Beobachten eines Unfalls einer anderen Person,
- betroffene Berufsgruppen: *Zugführer, medizinisches Personal, Angestellte des Einzelhandels und Lkw-Fahrer*,
- Ereignisse: Zeuge eines Suizids/Unfalltods eines anderen, Verkehrsunfälle, Beinahe-Tod-Erfahrung („in letzter Sekunde gerettet“), Raubüberfall,
- vorwiegend Störungsbilder der *akuten Belastungsreaktion oder PTBS*, nur vereinzelt komplexere Störungsbilder,
- meist zügige Überweisung nach einem bis 2 Monaten mit *rascher Besserung der Beschwerden*; im Durchschnitt wurden 10 Sitzungen benötigt (65 % ≤ 5 und 82 % ≤ 15 Sitzungen).

#### Infobox 1 Fallbeispiel

*Ereignis*: Ein 58-jähriger Lkw-Fahrer wurde in der Nacht stehend von einem von vorn kommenden Pkw bei Glatteis frontal gerammt. Der Unfallgegner ist dabei tödlich verunglückt. Der Versicherte blieb unverletzt.

*Symptomatik*: depressiv-ängstliche Verfassung, Flashbacks, Schlafstörungen mit wiederkehrenden Alpträumen („Den Blick des Fahrers, von Todesangst gezeichnet, werde ich nie vergessen.“), Angst und Schweißausbrüche beim Autofahren.

*Diagnose*: posttraumatische Belastungsstörung mit reaktiver Depression.

*Therapie und Verlauf*: psychiatrische Erstvorstellung nach 43 Tagen. Der Versicherte konnte seine Tätigkeit als Lkw-Fahrer aufgrund der Angststörung nicht wieder aufnehmen. Als selbstständiger Mietfahrer bestanden große finanzielle Sorgen, wodurch eine reaktive Depression bedingt war. Im Verlauf neue Existenz mit Baufirma aufgebaut, Beschwerden besserten sich dann deutlich. Nach 6 Sitzungen konnte die Behandlung beendet werden.

### Psychische Gesundheitsstörungen bei Schwerstverletzten

- Zuordnung bei rund 13 % der Kohorte,
- langwierige stationäre Aufenthalte, oft mehrfache Operationen und Rehamaßnahmen über mehrere Monate bis Jahre hinweg,
- somit auch sehr *späte Überweisung zur psychiatrisch-psychotherapeutischen Behandlung mit einer Latenz von meist >* *1 Jahr,*
- *anhaltende körperliche Leistungseinschränkungen und/oder chronische Schmerzstörungen* im Zusammenhang mit den Verletzungen.
- Alle Fälle zeigten ein *komplexes psychiatrisches Störungsbild mit meist begleitenden depressiven Symptomen*, teils auch Alkoholmissbrauch, chronischer Schmerz- und Persönlichkeitsstörung.
- Bis auf einen Patienten konnten alle ihren *Beruf nicht wieder aufnehmen* und wurden teilweise berentet.
- *Im Durchschnitt sind 15 Sitzungen* erfolgt.

#### Infobox 2 Fallbeispiel

*Ereignis*: Eine 63-jährige Pflegehelferin stürzte auf dem Heimweg mit dem Fahrrad und erlitt ein schweres Polytrauma (u. a. SAB, Pneumozephalus, subkapsuläre Leberruptur, Nebenniereneinblutung, Frakturen des Mittelgesichts, Kiefers, Nasenbeines und des Orbitabodens mit Durchtrennung des N. abducens). Mehrere Operationen notwendig mit langem stationärem Aufenthalt.

*Symptomatik*: komplette retrograde Amnesie, panische Angst vor dem Fahrradfahren, Affektlabilität, Schlafstörungen, vermehrter Alkoholkonsum.

*Diagnose*: Anpassungsstörung, Alkoholabusus.

*Therapie und Verlauf*: psychiatrische Erstvorstellung nach 163 Tagen. Insgesamt langwierige psychiatrische Therapie mit 25 Sitzungen. Einleitung einer Psychopharmakotherapie. Im Verlauf Besserung der Verfassung, Berentung, alkoholabstinent, erfolgreiches stufenweises Heranführen ans Fahrradfahren.

### Körperliche Verletzungen mit psychischen Symptomen

- Zuordnung bei rund 19 % der Kohorte.
- Zwei Drittel der Fälle waren in stationärer Behandlung.
- Im Durchschnitt erfolgte eine Überweisung nach < 3 Monaten.
- In 78 % der Fälle konnte die Therapie nach ≤ 5 Sitzungen beendet werden.
- Meist unkomplizierte Verläufe mit schneller Besserungstendenz.

### Fälle mit auffälligen Krankheitssymptomen

- Zuordnung bei der Minderheit (8 %) der Behandlungsfälle.
- Alle Patienten waren *im mittleren Alter* (im Durchschnitt 50 Jahre).
- Multifaktorielle Einflüsse für unerwartet langwierigen Verlauf – *meist schweres Trauma, eher spätere Überweisung* (im Mittel > 1 Jahr), *begleitende Rechtsstreitigkeiten* über Rentenanträge, Verletztengeld, Antrag auf Schwerbehinderung o. Ä.

### Fälle mit Belastungsfaktoren

- Zuordnung bei 25 % der Kohorte.
- In zwei Dritteln der Fälle waren *Konflikte mit dem Arbeitsgeber vordergründig* (u. a. Kündigung und generelle Unzufriedenheit im Beruf).
- In je einem Drittel der Fälle *familiäre Belastungen* (Erkrankungen, Probleme in der Partnerschaft) und *Traumareaktivierung von früheren Erlebnissen* (wie Gewalterfahrungen oder frühere Unfälle).

Das Kollektiv der Güstrower Klinik verdeutlicht exemplarisch die Variabilität der psychischen Folgestörungen und unterschiedlichen Behandlungsverläufe nach einem Arbeits- oder Wegeunfall. Die vorgeschlagene Typologie der DGUV erscheint auch in der klinischen Anwendung nützlich.

## Fazit

Das Psychotherapeutenverfahren der DGUV hat sich in den 10 Jahren ihres Bestehens etabliert, erkennbar an einer Verdoppelung von einem halben auf ein ganzes Prozent psychotherapeutisch mitbehandelter Fälle sowie dem deutlichen Ausbau des Psychotherapeutennetzwerks. Dabei erscheint in der Mehrheit der Behandlungsfälle eine Kurzzeittherapie ausreichend, um eine erfolgreiche psychotherapeutische Unterstützung und Behandlung zu leisten. Der tatsächliche Bedarf an Psychotherapie dürfte jedoch noch deutlich höher liegen, allerdings fehlen populationsrepräsentative Studien hierzu. Nach wie vor ist der D‑Arzt in der Mehrzahl der Fälle nicht derjenige, der die Mitbehandlung initiiert. Er ist aber Hinweisgeber durch entsprechende Angaben im D‑Arzt-Bericht bzw. im Verlaufsbericht. Weitere Aufklärung und spezifische Fortbildungen über die Komplexität psychischer Unfallfolgen sowie therapeutischer Möglichkeiten, einschließlich des Psychotherapeutenverfahrens, sind notwendig, um auch von D‑ärztlicher Seite ein aktiveres Überweisungsverhalten zu erreichen und eine bedarfsgerechte frühzeitige Therapie zu garantieren.

## Fazit für die Praxis

- Das Mitte 2012 eingeführte Psychotherapeutenverfahren der Deutschen Gesetzlichen Unfallversicherung (DGUV) wird von D‑ärztlicher sowie psychotherapeutischer Seite und nicht zuletzt von den Versicherten selbst zunehmend gut angenommen.
- Aktuell werden 1 % der beruflich Verunfallten psychotherapeutisch mitbehandelt. Der Prozentsatz psychischer Unfallfolgen liegt nach Schätzungen jedoch etwa 10-mal so hoch – eine populationsrepräsentative Studie fehlt hierzu.
- Psychoreaktive Störungen sind zumeist selbstregulierend und nur kurzzeitig andauernd.
- Auf psychische Warnhinweise nach einem Unfalltrauma ist zu achten. Eine spezifische Diagnostik ist bei Verdacht auf eine Traumafolgestörung frühzeitig einzuleiten.
- Es bedarf weiterer Aufklärung und unfallchirurgischer Fortbildungen über relevante Fallkonstellationen, Psychotraumatologie, sowie das Psychotherapeutenverfahren, um die Indikationsstellung für Psychotherapie zu verbessern.
